# PEDIATRICIANS’ REPRESENTATIONS ON DAIRY ALTERNATIVES WHEN WEANING IS UNAVOIDABLE

**DOI:** 10.1590/1984-0462/;2017;35;1;00007

**Published:** 2017

**Authors:** Vicente Sarubbi, Camila Junqueira Muylaert, Isabella Teixeira Bastos, Paulo Rogério Gallo, Claudio Leone

**Affiliations:** a Faculdade de Saúde Pública, Universidade de São Paulo (USP), São Paulo, SP, Brasil.

**Keywords:** Physicians, Pediatrics, Infant formula, Cow’s milk, Weaning

## Abstract

**Objective:**

To analyze pediatricians’ representations on the nutritional alternatives that are adopted when weaning becomes inevitable.

**Methods:**

This is a mixed cross-sectional analytical study with probabilistic sampling. Fifty-seven randomly selected pediatricians were interviewed with the use of a semi-structured script for thematic analysis. The technique of free evocations was used, and the terms were processed using software EVOC 2005. The thematic categories were established on software NVivo10, and their co-occurrence matrix was exported and analyzed in terms of their simple similarity hierarchy on software CHIC.

**Results:**

In the pediatricians’ representations, whole milk was cited as a foodstuff with high allergenic risk (35.1%) and nutritionally inappropriate, and they did not recommend its use if weaning occurred before 1 year of age. The infant formula, referred by 98.3% of the pediatricians as the best alternative at the moment of weaning, was cited by 38.1% of them owing to its nutritional adequacy. The points quoted as unfavorable to the use of the formula were the price, the possibility of causing allergy and the risk of the inadequate use of such a highly industrialized product.

**Conclusions:**

The pediatricians’ representations show that they are sensitive to the importance of breast-feeding and at the same time, to the sociocultural difficulties inherent in the practice. Generally speaking, the interviewed pediatricians recommend the use of milk formulas, and not of whole cow’s milk, if weaning occurs before the end of the first year of life.

## RESUMO

**Objetivo:**

Analisar as representações de pediatras sobre as alternativas alimentares adotadas quando o desmame se torna inevitável.

**Métodos:**

Estudo transversal qualiquantitativo, analítico e com amostragem probabilística. Foram sorteados 57 pediatras, que participaram de uma entrevista com o uso de um roteiro semiestruturado para a análise temática. Foi utilizada a técnica de evocações livres, e os termos foram processados pelos *softwares* EVOC 2005. Estabeleceram-se as categorias temáticas no *software* NVivo 10, e sua matriz de coocorrência foi exportada e analisada pela hierarquia de similaridade simples no *software* CHIC.

**Resultados:**

Nas representações dos pediatras, o leite integral foi evocado como um alimento com alto risco alergênico (35,1%) e nutricionalmente inadequado, e não se recomenda seu uso quando o desmame ocorre abaixo de 1 ano de idade. A fórmula infantil, referida por 98,3% dos pediatras como melhor opção no momento do desmame, foi evocada por 38,1%, em função de sua adequação de nutrientes. Os pontos desfavoráveis para o emprego da fórmula foram: o preço, a possibilidade de alergia e o risco da falta de critério na utilização de um produto altamente industrializado.

**Conclusões:**

As representações dos pediatras sugerem que eles estão sensíveis à importância do aleitamento materno e, ao mesmo tempo, às dificuldades socioculturais inerentes a essa prática. De modo geral, os pediatras entrevistados orientam o uso de fórmulas lácteas, e não o leite de vaca integral, se o desmame ocorre antes do fim do primeiro ano de vida.

## INTRODUCTION

In addition to the possibilities of mortality during childhood and school age,^1^ ^,^ ^2^ ^,^ ^3^ ^,^ ^4^ ^,^ ^5^ ^,^ ^6^ ^,^ ^7^ ^,^ ^8^ the first thousand days of life are considered crucial in determining risks for chronic noncommunicable diseases (NCDs). In this sense, growth and nutrition, both in the intrauterine period and early in life, have been shown to be associated with early risk of overweight and obesity.^9^ ^,^ ^10^

Prolonged breastfeeding, a protection factor against the development of excess weight in children, has not been a widespread practice, especially in urban areas.^11^ Reasons related to urban life, the mother’s working conditions, access to health services, and prematurity are reasons for early weaning.

In recent decades, many infant formulas have been developed and adapted to the needs of low-age children. To that end, qualitative and quantitative changes in sugars, fats, and mainly proteins were prepared, as well as changes in the caloric intake and the inclusion of active ingredients similar to those in breast milk. Though incomparable to breast milk, formulas represent an advancement in children’s feeding when compared to the use of whole cow’s milk.^12^ ^,^ ^13^

The hypothesis that based this study is that a high proportion of pediatricians have used, for various reasons, whole cow’s milk as the main choice in weaning.^14^ ^,^ ^15^ This study aimed to analyze the representations of pediatricians on food choices adopted at a time when weaning is inevitable and how they base their choices.

## METHOD

This is a cross-sectional, quantitative and qualitative (mixed study), and analytical study. The composition of the sample was random and stratified. All pediatricians regularly enrolled in the database of the Pediatrics Society of São Paulo were subjected to systematic random selection with replacement, and inclusion criteria for participation in the survey was exercising professional activities in São Paulo. In total, 209 pediatricians were randomly selected. The final sample consisted of 57 professionals. There were no exclusion criteria for participation between those selected, except for refusal by the professional or impediment from participating in the interviews in the collection period (vacation, leave of absence, or three unsuccessful interview attempts).

For the establishment of a qualitatively representative field of professional experts in which it was possible to capture the variability of the discourse by different aspects that would interfere with the practical orientation, the time since graduation, subspecialty field and place of professional activity were surveyed.

The study included 57 pediatricians, individually interviewed between November 2013 and June 2014 by graduate student researchers. The instrument for the production of data was a pretested semistructured script. The interviews were recorded and transcribed. The script covered guiding questions from three thematic fields: difficulties to maintain breastfeeding, senses that underlie the clinical managements on the weaning, and feeding low-age children.

From these key points, this study focuses primarily on the practices adopted at the time of weaning. Aside from the interviews, the free evocation of words technique was used with the inducer terms “whole milk,” “food for babies,” and “formulas,” which consists in asking the respondent what words come to their mind in relation to the inducer terms presented. The evocations are arranged in the order and frequency of appearance, building a body of key expressions for the analysis of representations of a particular social group.^16^ ^,^ ^17^ The terms evoked were processed by software EVOC 2005 and analyzed by frequency and by their position in the sequence mentioned, estimating the average evocation order (AEO).

The entry of the evocations in the Vergès analytical four-place chart was set as the value accumulated around 50% of all words mentioned (Table 1). The four places stress peripheral areas (first and second peripheries), the contrast and the central zone, having in mind the combination of frequencies and of each evoked term. The central tendency is expressed by the median evocations admitted in the chart, while, for the hierarchical value (rang) of the AEO, the mean of its weighted averages was calculated.^18^ ^,^ ^19^

**Table 1:**
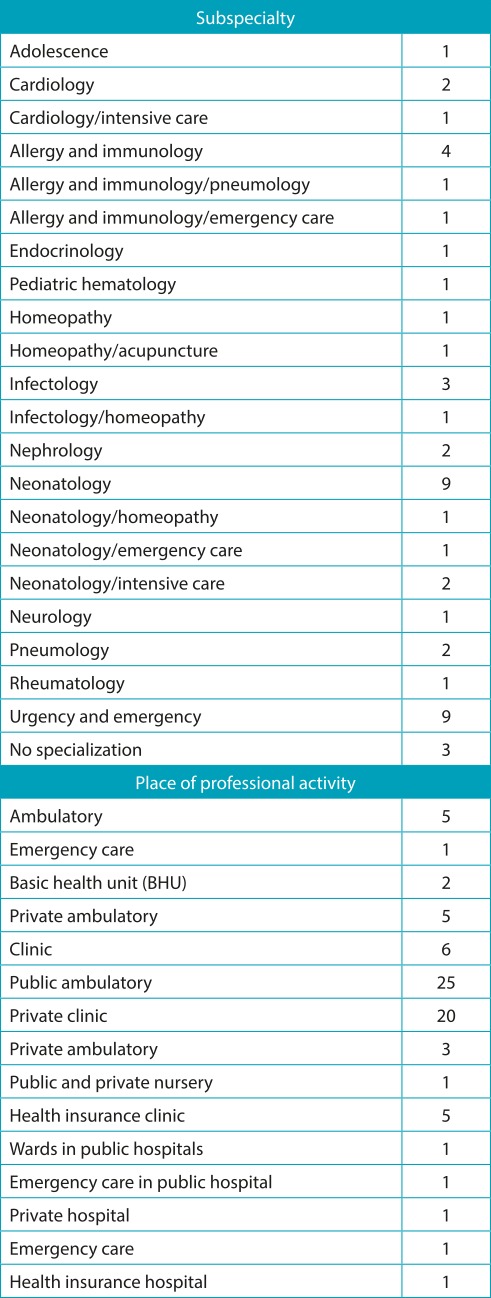
Distribution of pediatricians according to subspecialty and place of professional activity.

Evoked terms occupy a position of centrality in the discursive dynamics for having above the median and below the average frequency of the order in which the terms were evoked (AEO). In addition to the frequency, the lag time between the inducer stimulation and the recall was considered. It is accepted that the first four words and those more often evoked tend to represent associations strongly linked to feelings and the collective imagination. The contrast zone, although presenting evocations with low frequency, provides terms that are mentioned in the first positions, being thus of great importance to this small group of pediatricians. The peripheries refer to terms more closely related to the immediate context in which practices are undertaken.^18^ ^,^ ^19^ ^,^ ^20^

As for the themes of the answers collected, thematic-categorical content analysis technique was used.^21^ The thematic categories were processed in software NVivo 10,^22^ to identify the meanings of the discourse of professionals and how the thematic recurrences were distributed among respondents. The co-occurrence matrix generated was analyzed by the similarity hierarchy in software CHIC,^23^ to relate the different groups of associated thematic units.

The study was approved by the Research Ethics Committee of *Faculdade de Saúde Pública de São Paulo* at *Universidade de São Paulo* (USP), São Paulo Brazil, under protocol number 441.038/2014.

## RESULTS

About 80% of the 57 sampled pediatricians were aged between 30 and 60 years, mostly women (75%). Virtually all professionals had residency in pediatrics (98.2%). Forty-four of them had the title of experts (77%), and more than half had completed graduation over 15 years before (68.4%). Most pediatricians also acted in subspecialties, the most frequent being neonatology and emergencies. The predominant professional practice sites were public clinics and private practices (Table 1).

The characterization of free evocations, as proposed by Vergès (Table 2), for the inducer term “food for babies*”* points, in the first quadrant (centrality), that breast milk is the most significant term. In quadrants corresponding to the higher frequency areas, there is the adequacy of nutrients (type and/or quality of the food) as the term that highlights the importance of food for growth and healthy development of the baby.

**Table 2:**
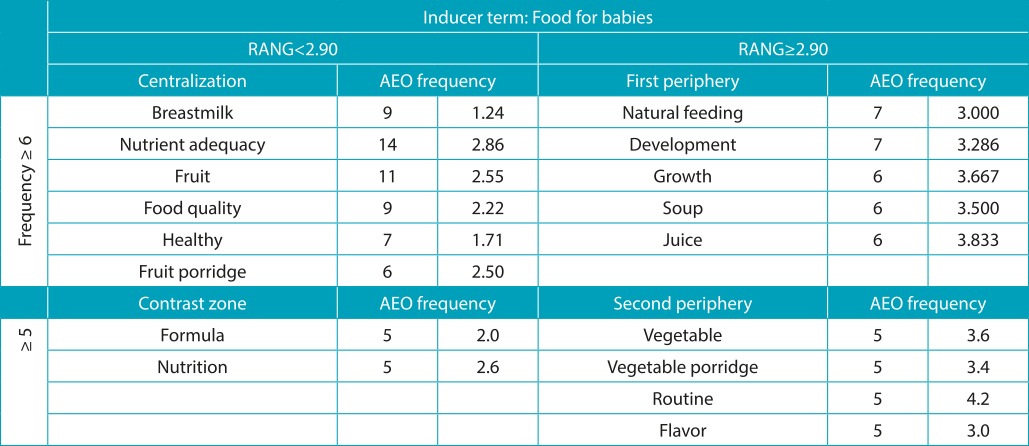
Vergès distribution of terms evoked by inducing stimuli *food for babies*, whole milk and formula, divided by the median and the average order of evocation (AEO). São Paulo, 2014.

RANG: hierarchic value; AEO: average evocation order.

On the other hand, the terms obtained in the four places of the inductor terms for whole milk and infant formula stress harmful characteristics, such as the inadequacy of nutrients in food for babies. Whole milk was mentioned as a possible risk to the child developing an allergy to cow’s milk protein and/or as having inadequate nutrient intake and/or as an inappropriate source of nutrition in the broad sense. Infant formula was evoked as the foodstuff with higher nutrient adequacy and/or referred to as very practical in handling and preparation. The high cost and cross-allergy appear as the main terms that emphasize critical points on the use of formula (Tables 3 and 4).

**Table 3:**
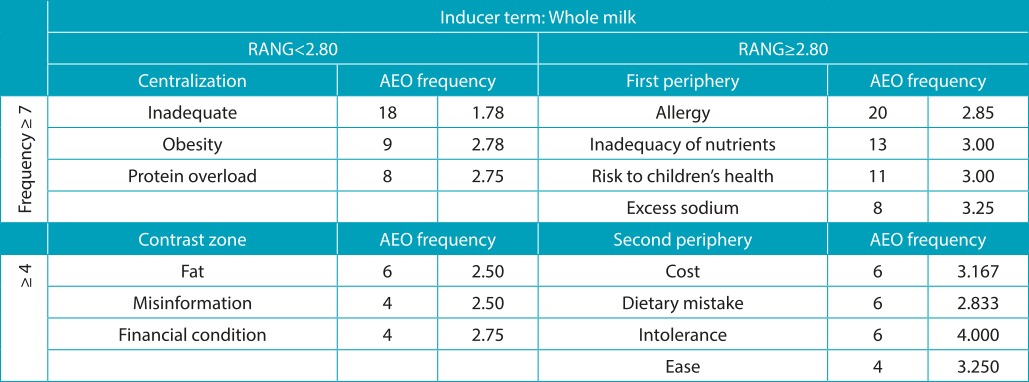
Vergès distribution of terms evoked by inducing stimulus *whole milk*, distributed by the median and the average order of evocation (AEO). São Paulo, 2014.

RANG: hierarchic value; AEO: average evocation order.

**Table 4:**
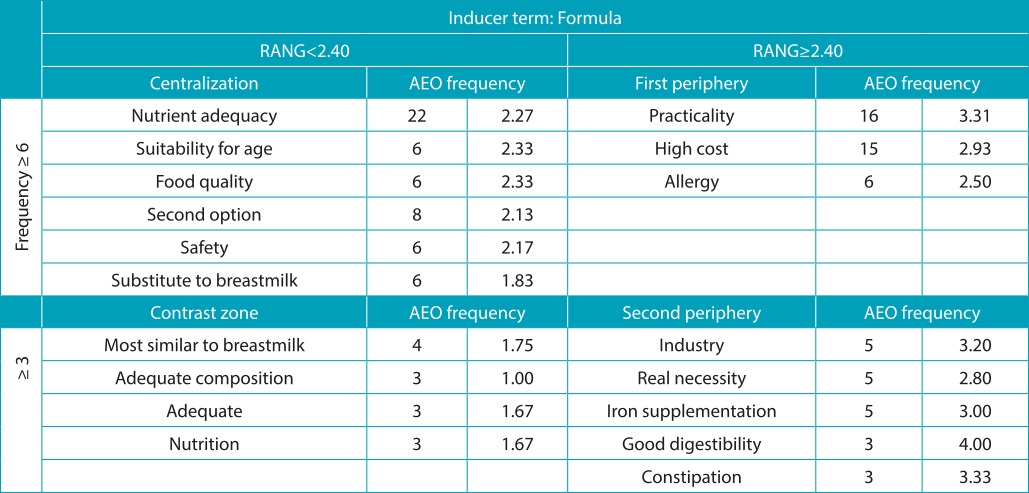
Vergès distribution of terms evoked by inducing stimulus *formula*, distributed by the median and the average order of evocation (AEO). São Paulo, 2014.

RANG: hierarchic value; AEO: average evocation order.

The exploration of the peripheries and contrast zones depending on the characteristics of pediatricians - their age, education time, subspecialty, and the place of professional activity - could not find controversial (conflicting/polarized) or emerging (own a subgroup) evocations that could characterize a specific subset of professionals that differed from a discourse that was rather reified by most respondents. The formula is referred to as a foodstuff that has adequate nutritional composition, and the most similar to breast milk, while whole milk is mentioned as a nutritionally inadequate food and its use is associated with misinformation and to the family’s financial condition.

With regard to the themes obtained from the content analysis, it is noted that the formula was recommended by 98.2% of pediatricians in case of weaning, early or not. It was also seen as the substance that best met the nutritional needs of the child for 33.3% of pediatricians. Family income was reported by 21.01% of pediatricians as a factor that interfered in the decision about the use of infant formula or in the family’s choice to keep the formula after 6 months of age.

In the analysis of similarity of the themes, it was found that the reports on the introduction of whole milk and other foods before the first year of life is often associated with family income. On the other hand, the formula appeared as the foodstuff that comes closest to breastmilk to better meet the nutritional needs of the child, the characteristic that most justifies the option for modified milk. The use of formula was also justified by scientific studies or by prescription, aiming to reduce allergies often more frequently than the references of the experience in the clinic (Table 5).

**Table 5:**
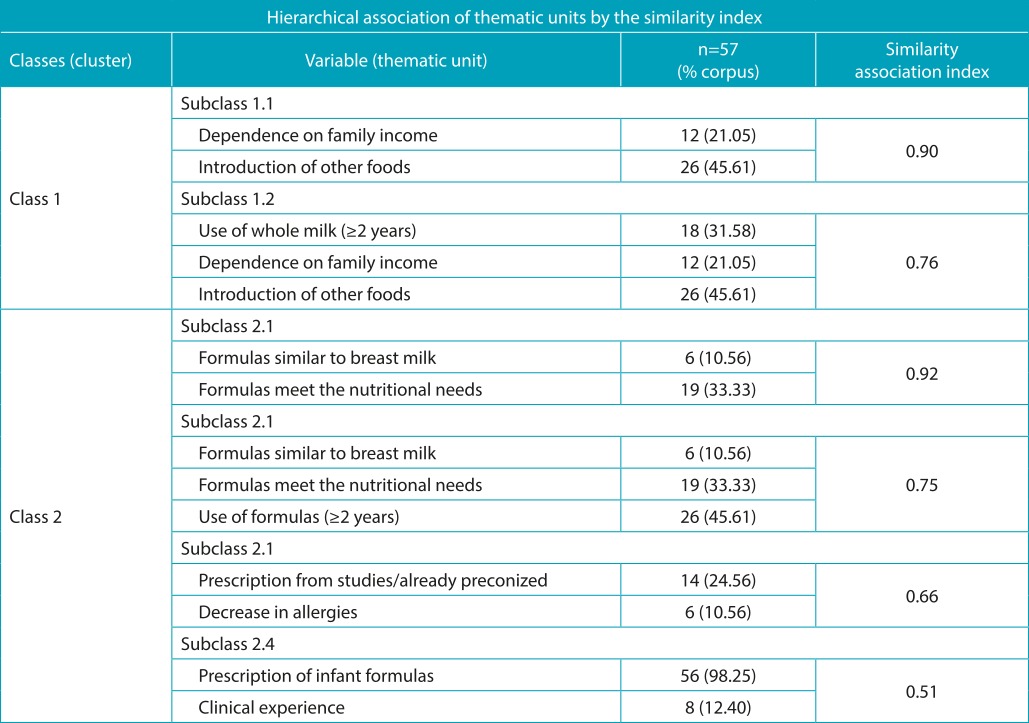
Hierarchical classification of similarity associations between the themes.

## DISCUSSION

Most reports show sensitivity to the importance of breastfeeding. Faced with the impossibility of its maintenance, pediatricians usually indicate the use of formulas modified from cow’s milk, even when they aim to resume breastfeeding, a situation in which the mixed feeding appears as an alternative when aiming to extend the duration of breastfeeding.

When the complete withdrawal of breast milk is an accomplished fact, pediatricians almost unanimously prescribe formulas as a strong ally in persuading the mother to not make use of whole cow’s milk:

*So, you keep on guiding, writing, telling the mother: look, up to six months, we will use the formula for zero to six months, then we will change it for one from six months to one year. You guide the mother. The brand does not matter, but it has to be from zero to six months, then six months to a year, because if you do not guide the mother and prescribe correctly, she goes out and buys cow’s milk.*

As also recommended by the American Academy of Pediatrics,^24^ the modified cow’s milk formula appears as an option for pediatricians before weaning and as an alternative associated with nutritional and allergenic restrictions. The use of soy-derived formulas appears as the last option when there are allergies to cow’s milk (even recognizing possible cross-allergies), or in cases where the family opts for a strict vegetarian diet.

According to pediatricians, the choice of the formula is related to the nutritional value, the composition nearest of that of breast milk, the characteristics of digestion and nutrient composition (proteins, particularly), characteristics necessary for the children’s different age groups, and potential benefits for development, with emphasis on neuromotor aspects.

*But I choose the formula because there is no other option, I know no other option. There is no use in diluting whole milk, as people did back then, which is not the same thing. You do not get the same amount of [docosahexaenoic acid] HDI and [arachidonic acid] ARA, micronutrients. You cannot introduce it as it was done before, diluting whole milk, and putting oil in it... You can no longer do this.*

The prescription related to clinical practice, reported by a smaller group of pediatricians, was brought as something learned in residency and more emphasized as an experience lived by professionals who had more time in private practice, though the guidance appeared more linked to preconization based on technical and scientific results than by the clinical experience of the pediatrician, regardless of their education time. This constitutes a highly consonant discourse in this group of pediatricians. Nevertheless, it was not possible to discriminate whether this knowledge comes from the dissemination between pairs or communications at scientific events, or from research developed by the food industry. The acceptance and safety in the prescription of infant formula, widely anchored in studies, and health protocols, make the product of the incorporated knowledge result in representations, such as allusion to its nutrient content, which now exerts a strong presence in professional conduct.^17^ ^,^ ^25^

There was a critical positioning regarding the use of formulas that were inappropriate - in quality or quantity - to the baby’s needs: the lack of guidance to the mother while still in the maternity regarding breastfeeding and early introduction of supplements, allergy risk the and idea of the mother and health professionals that the formula is a perfect substitute for breast milk. Other issues involved in early supplementation are linked to the demand for convenience among the family’s needs, to the ease of access to formulas and especially to the mother’s return to the labor market.

*Just as antibiotics are prescribed and the prescriptions are retained, if this kind of foodstuff was more controlled, it would lead to less errors like this, of parents using it with no information whatsoever. The difficulty of purchase would stimulate breastfeeding.*

With regard to the ease of access to the formulas, the study by Bunik et al.^26^ corroborates the results in this study by finding that mothers make the decision of supplement feeding with formulas, even without medical guidance, recommending a more restrictive distribution of formulas in hospitals:

*Regarding the guidance of a professional, from what I see today, you don’t have so many professional geared to keep this guidance focused on breastfeeding in favor of the difficulties.*

Other studies have shown better results in the support for breastfeeding from the support and proper guidance of doctors, family, friends and the work environment, as well as an education directed to the entire health team, since maternal difficulties breastfeeding can include the physical pain, the emotional pain, and discomfort underlying the expectations of breastfeeding, even among experienced mothers.^27^ ^,^ ^28^

In this study, the high cost of formulas also appears as a limiting factor for their use, although, for most pediatricians, benefits from the use of the formula overcome this issue, when the formula is compared to whole milk:

*We understand the financial difficulty, but when comparing the risk-benefit ratio for the child, I always try to prescribe formulas.*

However, even when the idea of adopting the infant formula facing weaning is consolidated for the professionals interviewed, family income appears in some reports as the most deterrent element for its indication or maintenance.

Starting from the second year of life, the indication of whole milk or formula appears to be quite diverse. Although there is a predominance of the indication of follow-up formulas in the second year, there was no consensus on the relevance of using whole milk or formulas, even if there is still concern about the protein overload, excess sodium and the risk of obesity due to the consumption of whole cow’s milk.^29^

It should also be noted that some pediatricians emphasize the need for the distribution of formulas in the public health network instead of whole milk, since it has already been established that formulas are the most suitable foodstuff facing weaning or during mixed feeding. This characterizes the iniquity, since only part of the population, more privileged, may have access to this feature, as established by the Convention on the Rights of the Child of the United Nations (UN):^30^

*It would greatly improve the lives of the population, introducing the formulas in the public health network. Due to the cost of these formulas, only part of the population has access to it.*

Another small group, although favorable to the prescription formulas, took a more cautious position regarding the incentive to its distribution and supervision, due to it being a highly industrialized product. In this regard, one professional was totally unfavorable to the use of formula.

It is worth mentioning that this study, with a quantitative and qualitative design (mixed) and random sampling, although having a sample that shows limitations regarding generalization, innovates both by its way of offering different methods of analysis that enable the comparison of the results described to confirm, refute, or produce new research hypotheses, and in the scientific disclosure of representations that guide pediatricians’ decisions, which will impact the family context and the growth and development of an individual with implications beyond childhood.

It can be concluded that dietary guidance based on what is recommended in the studies and child care protocols is a very cohesive discourse of pediatricians regarding the nutritional indication of formulas. Representations of pediatricians suggest that they are sensitive to the importance of breastfeeding and, at the same time, at the same time, to the current sociocultural difficulties inherent in this practice. In general, respondent pediatricians indicate the use of infant formula, and not whole cow’s milk, when weaning occurs before the end of the first year of life.
